# Acupuncture and Auricular Acupressure in Relieving Menopausal Hot Flashes of Bilaterally Ovariectomized Chinese Women: A Randomized Controlled Trial

**DOI:** 10.1093/ecam/nep001

**Published:** 2010-10-19

**Authors:** Jue Zhou, Fan Qu, Xisheng Sang, Xiaotong Wang, Rui Nan

**Affiliations:** ^1^Sino-Britain Joint Laboratory, College of Pharmaceutical Sciences, Zhejiang University, Hangzhou, Zhejiang 310058, China; ^2^Women's Hospital, School of Medicine, Zhejiang University, Hangzhou, Zhejiang 310006, China; ^3^Heilongjiang University of Chinese Medicine, Harbin, Heilongjiang 150040, China; ^4^The First Affiliated Hospital, Liaoning University of Chinese Medicine, Shenyang, Liaoning 110032, China; ^5^Colorado School of Traditional Chinese Medicine, 1441 York Street, Denver, CO 80206, USA

## Abstract

The objective of this study is to explore the effects of acupuncture and auricular acupressure in relieving menopausal hot flashes of bilaterally ovariectomized Chinese women. Between May 2006 and March 2008, 46 bilaterally ovariectomized Chinese women were randomized into an acupuncture and auricular acupressure group (*n* = 21) and a hormone replacement therapy (HRT) group (Tibolone, *n* = 25). Each patient was given a standard daily log and was required to record the frequency and severity of hot flashes and side effects of the treatment felt daily, from 1 week before the treatment started to the fourth week after the treatment ended. The serum levels of follicle stimulating hormone (FSH), LH and E_2_ were detected before and after the treatment. After the treatment and the follow-up, both the severity and frequency of hot flashes in the two groups were relieved significantly when compared with pre-treatment (*P* <  .05). There was no significant difference in the severity of hot flashes between them after treatment (*P* >  .05), while after the follow-up, the severity of hot flashes in the HRT group was alleviated more. After the treatment and the follow-up, the frequency of menopausal hot flashes in the HRT group was reduced more (*P* <  .05). After treatment, the levels of FSH decreased significantly and the levels of E_2_ increased significantly in both groups (*P* <  .05), and they changed more in the HRT group (*P* <  .05). Acupuncture and auricular acupressure can be used as alternative treatments to relieve menopausal hot flashes for those bilaterally ovariectomized women who are unable or unwilling to receive HRT.

## 1. Introduction

Hot flashes occur in the vast majority of post-menopausal women [1]. An extensive questionnaire study of 506 women found that 87% had daily hot flashes [2]. Hot flashes are episodic and usually accompanied by nausea, dizziness, headache, palpitations, diaphoresis or night sweats [3]. Having hot flashes may decrease a woman's quality of life by decreasing the quality of sleep and aggravating fatigue and depression [4, 5]. Menopausal hot flashes make most of women seek medical care during the menopausal transition [2]. Menopausal hot flashes are related to a psychological or mental disorder in menopausal women and the hormonal changes in these women may be the underlying mechanism [6, 7].

For those bilaterally ovariectomized pre-menopausal women, the estradiol (E_2_) contents in their serum were reduced by 80% [8]. The abrupt decline in E_2_ usually leads to more frequent and severe menopausal symptoms, especially hot flashes. Menopausal hot flashes are also related to enhanced norepinephrine (NE) activity in the hypothalamus, resulting in an abrupt, transient, downward resetting of the normal thermoregulatory response set point [9, 10]. Most of the bilaterally ovariectomized Chinese women have difficulty in stopping hormone replacement therapy (HRT) due to the severe menopausal symptoms. Although HRT historically has been used as the standard treatment for hot flashes [11], many women choose not to initiate or adhere to HRT because of its potential health risks and side effects [12, 13]. In recent years, non-pharmacological alternative treatments are being requested by more bilaterally ovariectomized women to relieve their menopausal symptoms, especially menopausal hot flashes. A study has demonstrated that acupuncture could induce accumulation of vaginal exfoliative cells, increase the weight of adrenal, and raise the level of serum corticosterone in ovariectomized model rats [14]. It is deduced that estrogen levels may be elevated using acupuncture to create compensatory hyperplasia of the adrenal cortex, thereby enhancing the transferring of androgen into estrogen in peripheral tissues [14]. In as early as 1995, the acupuncture's efficacy in relieving menopausal symptoms has been demonstrated [15]. Alternative and complementary therapies, including acupuncture, have been used increasingly in recent years to relieve menopausal symptoms [16–23], although one of them suggested that the used medical acupuncture was not any more effective for reducing menopausal hot flashes than was the chosen sham acupuncture [16]. As hot flashes are the most common menopausal symptoms in bilaterally ovariectomized women, it is important to find effective, non-pharmacological treatments to relieve their menopausal hot flashes. This study was designed to explore the effects of acupuncture and auricular acupressure in relieving menopausal hot flashes of bilaterally ovariectomized Chinese women.

## 2. Subjects and Methods

### 2.1. Subjects

Between May 2006 and March 2008, 46 bilaterally ovariectomized Chinese women were recruited through advertisement to complete 12 weeks of intervention with either acupuncture and auricular acupressure or Livial (Tibolone).

Inclusion criteria were as follows: the subject had received a bilateral ovariectomy in the previous 2 years and suffered from menopausal hot flashes; the patient did not manifest any perimenopausal symptoms before the bilateral ovariectomy and had not taken any drugs containing hormones or affecting the cardiovascular system during the previous 6 months; the level of thyroid-stimulating hormone (TSH) was normal and the E_2_ concentration was <50 pg/mL; a gynecologic examination and laboratory tests showed that the patient did not suffer from other organic diseases of the reproductive system after the bilateral ovariectomy; written consent was obtained from the subject stating that the subject would complete the study. To have been considered for the study, patients must have met all criteria.

Exclusion criteria were as follows: the subject was under other medical treatment during the research period, had metabolic, renal, anaphylactic or endocrine disease, or suffered from primary hypertension, primary hypotension, chronic anemia, tuberculosis, a mental disorder or a chronic affection; the body mass index (BMI) of the subject was more than 24 or she was a cigarette smoker. Patients were excluded from the study if they fit any of the above criteria.

The women were informed of the short- and long-term benefits of HRT and were informed about the aim and methodology of the study. Ethical approval and permission to conduct the study were obtained from the local ethical committee and the administration of the study was based on international ethical guidelines. Voluntary participation was requested and informed consent was obtained.

**Figure 1:**
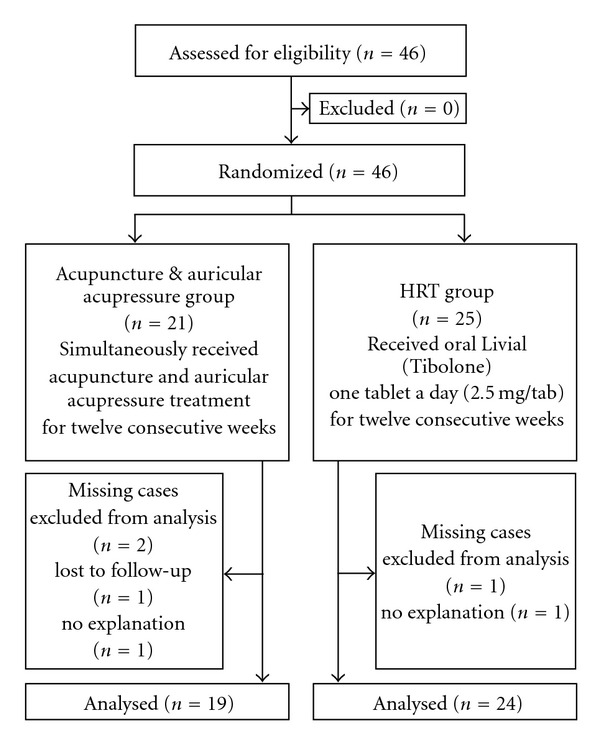
Study design.

Subjects were randomized to either the acupuncture and auricular acupressure group or the HRT group with the use of a randomization chart constructed in Microsoft Excel that randomized numbers into two groups. Having been divided, the acupuncture and auricular acupressure group had 21 cases and HRT group had 25. In the statistical analysis, 43 of the women were included. Three subjects were considered missing cases during the study (Figure 1). There was no significant difference in baseline characteristics between the two groups (Table 1).

Each patient was given a standard daily log and was required to record the frequency and severity of hot flashes and side effects of the treatment felt daily, from 1 week before the treatment started to the fourth week after the treatment ended. The patients were required to record the items before going to bed in the evening. The standard daily log was made in a structured way by the hospitals and all the possible side effects of the treatment had been listed on it, which had been validated beforehand.

Each participant received a physical examination, a routine blood examination, a routine uronoscopy, a liver function test and a renal function test, respectively, 1 day before the treatment started and 1 day after the treatment ended.

### 2.2. Group and Administration

#### 2.2.1. Acupuncture and Auricular Acupressure Group

Each patient randomized into the acupuncture and auricular acupressure group received both acupuncture and auricular acupressure treatments.

**Acupuncture**

The selected acupoints: Sanyinjiao (SP6), Fengchi (GB20), Hegu (LI4), Quchi (LI11), Guanyuan (CV4), Dazhui (GV14), Fuliu (KI7) and Zigong (EX-CA1).

The patient was in a comfortable, supine position. After the skin was routinely disinfected and the acupoints were carefully localized, filiform 0.35 mm × 40 mm sterilized, disposable needles (provided by Suzhou Hua Tuo Medical Instruments Co. Ltd, Suzhou, China) were inserted using the double hand-needle insertion technique. The depth of insertion was adjusted based on the patient's body size and the permissible depth of insertion of the specific acupoint. A technique using lifting, thrusting and twirling in a small range was performed until the appearance of De-qi, an obtained needle sensation, when there emerge a soreness, numbness and a feeling of distension around the point after the needle is inserted to a certain depth, and tenseness around the needle felt by the operator. Then, an even reinforcing-reducing technique was applied. The needle was retained for 40 minutes each time and manipulated twice during that time using a twirling technique in a small range. The manipulation lasted 30 seconds for each acupoint. The acupuncture treatment consisted of two sessions each week for 12 consecutive weeks.

**Auricular Acupressure**

The selected auricular acupoints: sympathetic (AH6a), shenmen (TF4), adrenal gland (TG2p), subcortex (AT4), endocrine (CO18), kidney (CO10), heart (CO15) and liver (CO12).

After the above acupoints were sterilized with 75% alcohol, pieces of plaster with magnetic beads of proper size and good quality were stuck to the acupoints, which were then pressed slightly until the patient had an aching pain, numbness, distention and a warm sensation. The patients were asked to press the acupoints by themselves six times a day for a 3 minutes duration each time. It was explained that the strength of the pressing should make the local auricle congestive, flushed, hot and achy. The auricular acupressure was alternatively conducted on the two ears every 2 days. The plaster with magnetic beads was exchanged for a fresh set once a week.

The acupuncture process adhered to the Standards for Reporting Interventions in Controlled Trials of Acupuncture (STRICTA) criteria [24].

#### 2.2.2. HRT Group

The patients were prescribed with oral Livial (Tibolone, made by Nanjing Oujianong Pharmaceutical Company Limited, Nanjing, China), in the dosage of one tablet a day (2.5 mg/tablet) for 12 consecutive weeks.

One day before the treatment and at the end of the treatment, the levels of follicle stimulating hormone (FSH) and luteinizing hormone (LH) were measured with enzyme-linked immunosorbent assay (ELISA) and the levels of E_2_ were measured with double antibiotic ELISA. All the reagents were provided by Lianxing Biological Technology Company, Tianjin, China.

### 2.3. Index and Method

The severity of hot flashes was defined as follows: mild—a fleeting, warm sensation without sweating or disruption of normal activities; moderate—a warm sensation associated with sweating, and disruption of normal activities; severe—a hot sensation associated with sweating and the discontinuation of normal activities [16, 19, 23, 25, 26].

The score of the hot flash severity for a particular day is calculated by adding 1 × the number of mild hot flashes + 2 × the number of moderate hot flashes + 3 × the number of severe hot flashes [16, 19, 23, 25, 26].

The frequency of hot flashes is the total number of the mild, moderate and severe hot flashes occurred during 24 hours.

### 2.4. Data Analysis

Results were analyzed by an independent university statistician using Statistical Package for Social Sciences (SPSS 13.0 for Windows), a computer software. Non-parametric Mann-Whitney tests were used to analyze the inter-group and intra-group differences of the severity and frequency of menopausal hot flashes. Analysis of variance (ANOVA) was used to compare the inter-group and intra-group differences of the serum levels of FSH, LH and E_2_. A 5% significance level (*P* < .05) and two-tailed tests were used for all hypothesis tests. Ninety-five percent confidence intervals (CI) for the median differences between the acupuncture and auricular acupressure group and the HRT group were determined.

## 3. Results

### 3.1. The Baseline Characteristics

There were no significant difference between the two groups' baseline characteristics (*P* > *.05*) (Table 1).

### 3.2. The Severity of Hot Flashes

There was no significant difference between the two groups' severity of hot flashes before treatment. After the 12 weeks of treatment, both groups' severity of hot flashes decreased significantly (*P* < *.05*) with no significant difference between the groups (*P* > *.05*). The 4-week follow-up showed that both groups were alleviated significantly when compared with pre-treatment (*P* < *.05*) and HRT had greater effects (*P* < *.05*) (Table 2).

### 3.3. The Frequency of Hot Flashes

There was no significant difference between the two groups before treatment. After the 12 weeks of treatment, the frequency of the menopausal hot flashes in both groups were reduced significantly (*P* < *.05*) and HRT had greater effects (*P* < *.05*) (Table 2). The 4-week follow-up showed that the frequency of hot flashes in the two groups both decreased significantly when compared with pre-treatment (*P* > *.05*) and the patients in the HRT group were alleviated more (*P* < *.05*) (Table 2).

### 3.4. The Serum Levels of FSH, LH and E_2_

Before treatment, there was no significant difference in the serum levels of FSH, LH and E_2_ between the acupuncture and auricular acupressure group and the HRT group. After treatment, the levels of FSH decreased significantly and the levels of E_2_ increased significantly in both of the two groups and the levels of LH decreased significantly in the HRT group (*P* < *.05*) (Table 2). The serum levels of FSH, LH and E_2_ in the HRT group changed more (*P* < *.05*) (Table 2).

### 3.5. Adverse Result

No side-effect was reported in either group during the period of the research or in the follow-up phase.

## 4. Discussion

Although menopause is associated with changes in the hypothalamic and pituitary hormones that regulate the menstrual cycle, menopause is not a central event, but rather primary ovarian failure [27]. As the hypothalamic-pituitary-ovarian axis remains intact during the menopausal transition, FSH levels rise in response to ovarian failure and the absence of negative feedback from the ovary [27]. Atresia of the follicular apparatus, in particular the granulosa cells, results in the reduced production of estrogen and inhibin, which leads to the reduced inhibin levels and the elevated FSH levels, a cardinal sign of menopause [27]. Correlations between endocrine levels and symptom severity ratings over time revealed that hot flash severity was significantly and positively related to FSH [28]. Investigations of hormonal connections between hot flash severity and reproductive hormones in Study of Women's Health Across the Nation (SWAN), Melbourne Midlife Women's Health Project (MMWHP) and Penn Ovarian Aging Study cohorts found that decreased serum E_2_ and increased serum FSH were associated with the increases in hot flash severity [29–31]. In an analysis of SWAN data, which modeled the effects of FSH and E_2_ (and other reproductive hormones) together, Randolph and colleagues noted that FSH was associated with hot flash prevalence and frequency [7].

It is well known that acupuncture is associated with homeostatic regulation, and possess effects such as buffering hormonal disturbance, modulating ovulation, as well as improving psychological or behavioral abnormity [32–34]. Acupuncture in specific acupoints has been found to significantly increase blood concentrations of E_2_ in the ovariectomized rats [35], while reducing the elevated plasma LH due to ovariectomy [36]; in addition, acupuncture also restored the number of gonadotropin-releasing hormone (GnRH) neurons in the ovariectomized rats [35]. In another study, acupuncture was found to improve the reproductive disorders induced by ovariectomy in rats through modulating the blood E_2_ levels [37]. Acupuncture may improve the function of the hypothalamic-pituitary-ovarian axis, increase blood adrenogenous androgen level and facilitate its transformation into estrogen by aromatic enzyme in the brain, liver and fat tissues [38–40].

The present study showed that acupuncture and auricular acupressure significantly relieve the severity and frequency of menopausal hot flashes. The levels of FSH decreased significantly and the level of E_2_ increased significantly in both of the two groups after treatment. As the increased levels of FSH and the lowered level of E_2_ are mainly associated with hot flashes during the menopausal transition [29–31], it may be partly through decreasing the levels of FSH and increasing the levels of E_2_ that acupuncture and auricular acupressure alleviate the severity and frequency of menopausal hot flashes of the bilaterally ovariectomized Chinese women. In comparison with HRT, although acupuncture did not change hormone levels as significantly as HRT in this study, the bilaterally ovariectomized women's own functions may be regulated with the use of acupuncture, while HRT restores the body's hormone level by exogenous hormones.

The protocol of the acupuncture treatments comes from a combination of literatures and clinical experiences. There is no corresponding name to menopausal hot flashes in ancient books of traditional Chinese medicine (TCM). Based on Zang-fu organs in TCM, disorders of the kidney and liver are generally considered as the main pathogenesis. According to the principle of reinforcing the kidney and regulating the liver, the main acupoints selected were Sanyinjiao (SP6), Fengchi (GB20), Hegu (LI4), Quchi (LI11), Guanyuan (CV4), Dazhui (GV14), Fuliu (KI7) and Zigong (EX-CA1). Among them, Sanyinjiao (SP6), Fengchi (GB20), Hegu (LI4) and Quchi (LI11) have been selected as the main acupoints in most of the clinical researches on menopausal hot flashes [19, 23]. Sanyinjiao (SP6) is an important acupoint to treat disorders of the spleen, liver and kidney [41]. It is able to soften and harmonize the Liver and to benefit the kidney Qi, based on the ancient classics of acupuncture [42]. Fengchi (GB20) works to regulate the function of liver and remove heat from the head and eye, based on the ancient acupuncture records and the Meridian Theory of TCM [41]. Hegu (LI4) functions to clear heat in the Upper and Middle Jiao [41]. Quchi (LI11) has been acknowledged to produce hypothermia in normal subject [42]. Guanyuan (CV4) is an important acupoint on the Conception Vessel and functions to reinforce kidney Qi and replenish Qi and blood [41]. Sanyinjiao (SP6) and Guanyuan (CV4) have been found to increase the gonadal hormone levels [43]. Dazhui (GV14) has been found to produce hypothermia in normal adult [44]. Fuliu (KI7) can regulate kidney Qi and sweating [41]. Zigong (EX-CA1) is an important empirical one for treating female disorders [41].

According to the theory of TCM, all channels of the body and all 12 meridians are closely connected with the ear [45]. The earliest record of ear acupuncture is in *Huangdi Neijing (The Yellow Emperor*'*s Classic of Internal Medicine)*. In the famous Chinese medical classics, a great number of acupuncture treatments were summarized, among which, there are the specific acupuncture points on the external ear for relief of certain disorders. Auricular acupressure could associate the meridians of the body, regulate Qi and activate blood, regulate Zang-fu organs and promote good metabolism according to the Meridian Theory of TCM [45]. There are 200 acupoints on the outer ear. Auricular acupressure's effectiveness and non-invasiveness make it easily accepted by both patients and doctors [45, 46]. Auricular acupressure works by stimulating the central nervous system through the cranial and spinal nerves on the auricle of the ear. This stimulation results in increasing neurotransmitters within the pituitary and spinal cord of the central nervous system [45]. All the auricular acupoints selected in the study were associated with enforcing kidney Qi and regulating liver Qi.

Acupuncture has been found to significantly reduce the severity of nocturnal hot flashes in post-menopausal women [19]. Standardized and individually tailored acupuncture treatment was also found to significantly decrease the severity of hot flashes in symptomatic post-menopausal women when compared with placebo acupuncture of equal duration [23]. Another study showed that acupuncture and applied relaxation significantly reduced the number of menopausal hot flashes [22]. The present study showed that acupuncture and auricular acupressure significantly relieve the severity and frequency of menopausal hot flashes. However, as the sample size of the present study was small and the sham-acupuncture was not used, the conclusion may be somewhat limited.

Although the double-blind randomized controlled trial (RCT) has been known as the gold standard in clinical researches, the biggest challenges and the difficulties to the researchers in the fields of clinical acupuncture and moxibustion are the design of an ideal placebo-control method and the credibility and ethics behind sham acupuncture [47–52]. An ideal acupuncture placebo that avoids the necessity of penetration of the skin and shows the same psychological impact has not yet been found. A study has shown that for non-drug interventions including acupuncture, it was difficult to establish a placebo or sham control that is both inert and indistinguishable [53]. It concluded that although randomized trials investigating the specific effects of acupuncture have used a great variety of sham interventions as controls, the sham interventions as “placebo" controls seem misleading and scientifically unacceptable [53]. Another study demonstrated that the control interventions were equally as effective as acupuncture in alleviating pain in conditions that are predominantly associated with affective components such as migraines or lower back pain, but not those with a more pronounced sensory component, such as osteoarthritis of the knee or lateral epicondylalgia [54]. Some previous research also showed that nearly 40% of the participants in clinical research were able to detect a difference between the active and placebo needles at active points [55]. In a clinical study on the effect of acupuncture in treating post-menopausal hot flashes, although they combined the use of placebo needles with sham points in the placebo treatment, a difference in the patients' expectations of benefit was still found, and they considered finding an optimal placebo for acupuncture remained a challenge for future studies [23]. In addition, as most of the middle-aged Chinese people have had experiences receiving acupuncture treatments, it is even more difficult to simulate real acupuncture in clinical researches. This is why the study did not attempt to use sham-acupuncture as a control. Although this may introduce bias into trials, extensive details regarding the baseline characteristics of the recruited women were collected before treatment and between the two groups, no significant difference in the patients' detailed baseline characteristics existed. Further research with larger samples needs to be conducted.

## 5. Conclusions

Acupuncture and auricular acupressure can be used as alternative treatments to relieve menopausal hot flashes for those bilaterally ovariectomized women who are unable or unwilling to receive HRT.

## Funding

Natural Medicine Research UK (to J.Z.); China Postdoctoral Science Foundation (no. 20080441265 to J.Z.); Zhejiang Traditional Chinese Medicine Foundation (no. 2008YA015 to J.Z.); Zhejiang Province Postdoctoral Science Foundation (to J.Z.); China Postdoctoral Science Foundation (no. 20070421188 to F.Q.); Outstanding Young Medical Scientist Foundation of Zhejiang Province (no. 2008QN022); Zhejiang Traditional Chinese Medicine Foundation (no. 2008YB010).

## Figures and Tables

**Table 1:** The baseline characteristics of the participants.

Item	Acupuncture and auricular acupressure group (*n* = 19)	HRT group (*n* = 24)
*Demographic characteristics*		
Age (years)	41.6 ± 5.8	42.8 ± 4.1
Time since ovariectomy (years)	1.3 ± 0.4	1.2 ± 0.5
Married/living as married	14 (73.7%)	18 (75.0%)
Divorced/separated	4 (21.1%)	5 (20.8%)
Never married	1 (5.3%)	1 (4.2%)
Women with children	14 (73.7%)	14 (58.3%)

*Area of residence*		
Urban	12 (63.2%)	21 (87.5%)
Suburban	3 (15.8%)	2 (8.3%)
Small town	2 (10.5%)	0
Rural	2 (10.5%)	1 (4.2%)

*Current employment status*		
Full-time employment	8 (42.1%)	18 (75.0%)
Part-time employment	5 (26.3%)	2 (8.3%)
Registered unemployed	3 (15.8%)	2 (8.3%)
Retired	3 (15.8%)	2 (8.3%)

*Cause of ovariectomy*		
Bilateral tubal-ovarian cyst	2 (10.5%)	8 (33.3%)
Bilateral tubal-ovarian abscess	3 (15.8%)	2 (8.3%)
Malignant tumor of uterus and/or ovary	3 (15.8%)	2 (8.3%)
Benign tumor of uterus and/or ovary	2 (10.5%)	3 (12.5%)
Removing the need for contraception	1 (5.3%)	2 (8.3%)
Prophylactic ovariectomy	2 (10.5%)	1 (4.2%)
Bilateral severe ovarian endometriosis	3 (15.8%)	2 (8.3%)
Cessation of menstruation	2 (10.5%)	2 (8.3%)
Other	1 (5.3%)	2 (8.3%)
Duration of the menopausal hot flashes (year)	1.1 ± 0.5	1.3 ± 0.4

**Table 2:** The severity and frequency of hot flashes and the serum levels of FSH, LH and E_2_.

Item	Acupuncture and auricular acupressure group (*n* = 19)	HRT group (*n* = 24)
Hot flash severity		
Pre-treatment	14.71 ± 3.81 (95% CI 12.96–16.52)	15.28 ± 4.06 (95% CI 13.53–16.96)
Post-treatment	3.86 ± 0.84* (95% CI 3.40–4.45)	3.71 ± 0.65* (95% CI 3.44–3.97)
Follow-up	4.45 ± 0.90^∗,#^ (95% CI 3.92–4.95)	3.25 ± 0.34* (95% CI 3.00–3.33)

Hot flash frequency		
Pre-treatment	14.21 ± 2.42 (95% CI 13.37–15.88)	15.11 ± 4.96 (95% CI 13.04–17.30)
Post-treatment	10.32 ± 3.13^∗,#^ (95% CI 8.80–12.32)	7.48 ± 2.69* (95% CI 6.33–8.59)
Follow-up	7.69 ± 1.48^∗,#^ (95% CI 7.12–8.63)	5.58 ± 1.64* (95% CI 4.86–6.22)

FSH (mIU/mL)		
Pre-treatment	51.5 ± 13.9 (95% CI 46.7–58.7)	53.0. ± 14.3 (95% CI 46.9–59.0)
Post-treatment	40.4 ± 9.6^∗,#^ (95% CI 34.6–44.5)	22.7 ± 5.1* (95% CI 20.6–24.8)

LH (mIU/mL)		
Pre-treatment	33.7 ± 6.4 (95% CI 30.2–37.0)	32.9 ± 7.1 (95% CI 29.9–35.9)
Post-treatment	31.9 ± 5.7^#^ (95% CI 28.6–34.9)	15.6 ± 4.8* (95% CI 13.6–17.6)

E_2_ (pg/mL)		
Pre-treatment	37.1 ± 8.9 (95% CI 32.0–41.5)	39.4 ± 7.0 (95% CI 36.4–42.3)
Post-treatment	45.4 ± 7.3^∗,#^ (95% CI 41.7–49.4)	71.5 ± 13.4* (95% CI 65.8–77.1)

FSH, follicle stimulating hormone; LH, luteinizing hormone; E_2_, estradiol; CI, Confidence intervals. *P < *.05*, compared with pre-treatment in the same group. ^*#*^ *P* < *.05*, compared with the HRT group.
